# The Two Stem Cell MicroRNA Gene Clusters C19MC and miR-371-3 Are Activated by Specific Chromosomal Rearrangements in a Subgroup of Thyroid Adenomas

**DOI:** 10.1371/journal.pone.0009485

**Published:** 2010-03-03

**Authors:** Volkhard Rippe, Lea Dittberner, Verena N. Lorenz, Norbert Drieschner, Rolf Nimzyk, Wolfgang Sendt, Klaus Junker, Gazanfer Belge, Jörn Bullerdiek

**Affiliations:** 1 Center for Human Genetics, University of Bremen, Bremen, Germany; 2 Department of General and Visceral Surgery, St. Joseph Stift, Bremen, Germany; 3 Department of Pathology, Hospital Bremen-Mitte, Bremen, Germany; 4 Small Animal Clinic and Research Cluster of Excellence “REBIRTH”, University of Veterinary Medicine, Hanover, Germany; University of Barcelona, Spain

## Abstract

Thyroid adenomas are common benign human tumors with a high prevalence of about 5% of the adult population even in iodine sufficient areas. Rearrangements of chromosomal band 19q13.4 represent a frequent clonal cytogenetic deviation in these tumors making them the most frequent non-random chromosomal translocations in human epithelial tumors at all. Two microRNA (miRNA) gene clusters i.e. C19MC and miR-371-3 are located in close proximity to the breakpoint region of these chromosomal rearrangements and have been checked for a possible up-regulation due to the genomic alteration. In 4/5 cell lines established from thyroid adenomas with 19q13.4 rearrangements and 5/5 primary adenomas with that type of rearrangement both the C19MC and miR-371-3 cluster were found to be significantly overexpressed compared to controls lacking that particular chromosome abnormality. In the remaining cell line qRT-PCR revealed overexpression of members of the miR-371-3 cluster only which might be due to a deletion accompanying the chromosomal rearrangement in that case. In depth molecular characterization of the breakpoint in a cell line from one adenoma of this type reveals the existence of large Pol-II mRNA fragments as the most likely source of up-regulation of the C19MC cluster. The up-regulation of the clusters is likely to be causally associated with the pathogenesis of the corresponding tumors. Of note, the expression of miRNAs miR-520c and miR-373 is known to characterize stem cells and in terms of molecular oncology has been implicated in invasive growth of epithelial cells in vitro and in vivo thus allowing to delineate a distinct molecular subtype of thyroid adenomas. Besides thyroid adenomas rearrangements of 19q13.4 are frequently found in other human neoplasias as well, suggesting that activation of both clusters might be a more general phenomenon in human neoplasias.

## Introduction

**Figure 1:**
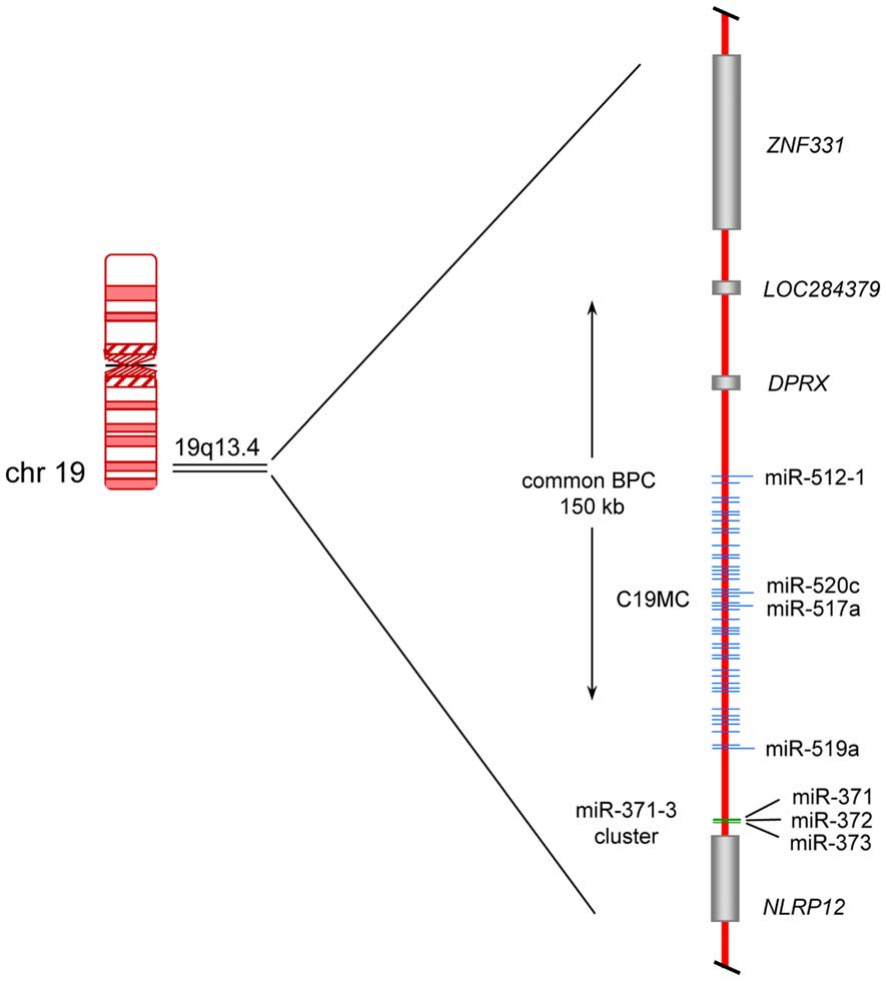
Scheme of the chromosomal region 19q13.4 with the two miRNA clusters C19MC and miR-371-3. Protein coding genes are represented by gray bars whereas genes of miRNA clusters are given as blue (C19MC cluster) and green (miR-371-3 cluster) lines, respectively. The common breakpoint cluster (BPC) of benign thyroid tumors of about 150 kb is indicated by a vertical arrow. miR-512-1 (pre-miR) is coding for mature-miR-512-5p, miR-371 (pre-miR) is coding for mature-miR371-3p. Gene symbols refer to the following protein coding genes: *ZNF331*  =  *zinc finger protein 331*, *DPRX*  =  *divergent-paired related homeobox*, *NLRP12*  =  *NLR family*, *pyrin domain containing 12.*

Thyroid adenomas are highly frequent human tumors that can be distinguished from their malignant counterparts i.e. follicular carcinomas by an encapsulated growth and a lack of invasiveness, respectively. Even in iodine sufficient areas thyroid adenomas occur in 4–7% of adults and in iodine deficient areas this number can rise to about 50%. The pathogenesis of these frequent benign tumors is only poorly understood but clonal chromosomal aberrations can be observed in roughly 40% of the nodules and are likely to pinpoint genomic regions and genes relevant for the development of the disease [1]. About 20% of the tumors with clonal cytogenetic aberrations show abnormalities involving chromosomal band 19q13 [2]. Given the extremely high prevalence of thyroid adenomas in Europe and the U.S. alone four to five million people can be estimated to be affected by this genomic alteration in their thyroid. So far, by positional cloning and *in silico* analyses the breakpoints have been found to cluster within a segment of 150 kb (kilobases) [3] that is located in close proximity to the genes encoding two miRNA clusters i.e. C19MC and miR-371-3 (Figure 1). The 100 kb long C19MC cluster with 46 tandemly repeated, primate-specific miRNA genes accounts for about 8% of all known human miRNA genes making it the largest human miRNA gene cluster discovered to date [4]. Ren et al. [5] have predicted 4,691 targets for this cluster. Recent evidence suggests that its miRNAs are encoded by an intron of a non-protein coding Pol-II transcript which is mainly expressed in the placenta [4]. In contrast to that large cluster the miR-371-3 cluster is much smaller spanning a region of approximately 1,050 bp where five miRNAs are encoded. The miRNAs of both clusters belong to a large miRNA family sharing a similar seed sequence [6]. Of note, several groups recently have linked the expression of members of the C19MC as well as the miR-371-3 cluster with the miRNA signature characteristic for human embryonic stem cells (hESC) [5], [6], [7]. First evidence for an oncogenic potential of miR-373 has been obtained in human testicular germ cell tumors where it was shown to allow tumorigenic growth in the presence of wild-type p53 [8]. In prostate cancer both miR-373 and miR-520c although found to be downregulated stimulated migration and invasion *in vitro* [9]. Recently, Huang et al. [10] were able to demonstrate that miR-373 and miR-520c promote tumor invasion and metastasis *in vivo* and *in vitro* by the suppression of CD44. Interestingly, qualitative and quantitative changes of CD44 expression have been implicated in the growth and progression of thyroid tumors. Because invasive behavior is of pivotal significance in the differential diagnosis of thyroid tumors we have addressed this study on a possible up-regulation of both miRNA clusters in thyroid adenomas.

## Results

### Cell Lines from Thyroid Adenomas with 19q13-Rearrangement Show Upregulated Expression of miRNAs of the C19MC Cluster

**Figure 2:**
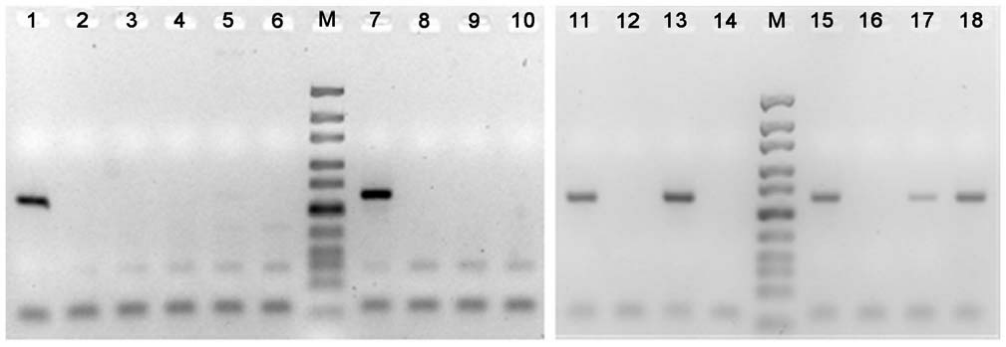
Expression analysis of miR-517a by RT-PCR. PCR reactions were performed and then analyzed in 4% small DNA Agarose. The expected DNA-fragment has a size of 62 bp, Ultra low range Ladder (Fermentas) was used as Marker (M). Lane 1: S40.2, 2: S40.2 without reverse transcriptase (–RT), 3: S121, 4: S121–RT, 5: thyroid (normal), 6: thyroid–RT, 7: placenta, 8: placenta-RT, 9: S270.2, 10: S270.2–RT, 11: S290.1, 12: S290.1–RT, 13: S141.2, 14: S325, 15: S211, 16: S211–RT, 17: fetal RNA, 18: adult testis, 19: fetal RNA-RT, 20: S141.2-RT, 21: adult testis-RT, 22: S325-RT (for details of the cell lines and tumor samples see Table 1).

To evaluate the role of either of the two miRNA clusters located in close proximity to the breakpoint region as possible targets of the 19q13 translocations in thyroid adenomas, we have first used RT-PCR to compare the expression of three members of the C19MC cluster, i.e. miR-512-5p, miR-517a, and miR-519a in five cell lines established from thyroid adenomas with 19q13 rearrangements and three cell lines from adenomas with other clonal abnormalities (Table 1). All cell lines had been established from primary tumors by using a SV40 derived subgenomic fragment. The three miRNAs chosen were spread over the whole cluster and served as examples for the more than 46 different but similar cluster members. Four of the five cell lines with 19q13 rearrangements expressed detectable levels of the three miRNAs whereas in the remaining cell line (S121, Table 1) and all cell lines with other aberrations no expression of any of the three miRNAs was noted (Figure 2).

**Table 1:** Used tissue samples and cell lines.

sample no.	thyroid material	cytogenetic subtype/FISH karyotype
S40.2*	cell line	46,XX,t(1;19)(p35 or p36;q13)[19]
S121*	cell line	46,XX,t(5;19)(q13;q13)[52]
S141.2*	cell line	46,XX,t(2;19)(p12 or p13;q13)[59]
S211^#^	cell line	46,XX,inv(4)(p15.2q12),t(5;19)(p14 or 15.1;q13),t(9;18)(q12;q22)[25]
S270.2	cell line	46,XX,t(2;3)(q21;q27 or q28)[13]
S290.1	cell line	46,XX,t(11;19)(q23;q13)[19]
S325^π^	cell line	46,XX,t(2;20;3)(p21;q11.2;p25)[17]
S533^π^	cell line	46,XX,t(2;7)(p21;p15)[16]
S805	adenoma	46,XX
S806	adenoma	46,XX
S889	adenoma	46,XX
S920	adenoma	46,XX
S925	adenoma	46,XX
S801	adenoma	46,XY,t(2;4),t(2;14;19)
		nuc ish(5′-tbpc19,3′-tbpc19)x2(5′-tbpc19 sep 3′-tbpc19x1)
S814	adenoma	46,XX,del(6)(q21∼22)
		nuc ish(5′-tbpc19,3′-tbpc19)x2(5′-tbpc19 sep 3′-tbpc19x1)
S842	adenoma	46,XX,t(1;19)(q32;q13)[8]/46,XX[24]
		nuc ish(5′-tbpc19,3′-tbpc19)x2(5′-tbpc19 sep 3′-tbpc19x1)
S846	adenoma	46,XY
		nuc ish(5′-tbpc19,3′-tbpc19)x2(5′-tbpc19 sep 3′-tbpc19x1)
S849	adenoma	not evaluable by cc
		nuc ish(5′-tbpc19,3′-tbpc19)x2(5′-tbpc19 sep 3′-tbpc19x1)

Cytogenetic details of the analyzed samples from follicular thyroid tumors and the cell lines used with their genetic subgroups determined by conventional cytogenetics and/or by interphase fluorescence in situ hybridization (I-FISH) with break-apart, dual-color rearrangement probe (tbpc-19). In case of the cell lines only the clonal aberrations found in the original tumors the cell lines have been established from are given.

Ref.: (_*_) [30]; (#) [2]; (π) [31].

**Figure 3:**
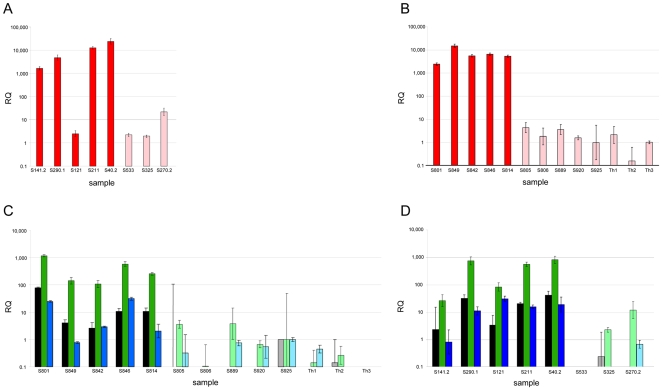
Expression of miR-520c, miR-371-3p, miR-372 and miR-373 in cell lines and primary tumors. Relative expression of miRNAs was determined by real-time PCR (mean s.d. from three independent experiments). Values of miRNA were normalized to *RNU6B (RNA, U6 small nuclear 2)* (**A**) miR-520c expression in thyroid cell lines, five cell lines derived from adenomas with 19q13.4 rearrangements (S141.2, S290.1, S121, S211, S40.2) (red bars) and three cell lines derived from thyroid adenomas with other structural rearrangements (S533, S325, S270.2) (light red bars). (**B**) miR-520c expression in three samples of non-neoplastic thyroid tissues (Th1, Th2, Th3) (light red bars), five adenomas with 19q13.4 rearrangement (S801, S849, S842, S846, S814) (red bars) and five adenomas without cytogenetically detectable aberrations (S805, S806, S889, S920, S925) (light red bars). (**C**) miR-371-3 expression in three samples of non-neoplastic thyroid tissues (gray (miR-371-3p), light green (miR-372) and light blue (miR-373)), five adenomas with 19q13.4 rearrangement (black (miR-371-3p), green (miR-372) and blue (miR-373)) and five adenomas without cytogenetically detectable aberrations (gray, light green and light blue bars) (for case numbers refer to Table 1). (**D**) miR-371-3 expression in thyroid cell lines, five cell lines derived from adenomas with 19q13.4 rearrangements (black (miR-371-3p), green (miR-372) and blue (miR-373)) and three cell lines derived from thyroid adenomas with other structural rearrangements (gray (miR-371-3p), light green (miR-372) and light blue (miR-373)) (for case numbers refer to Table 1).

We have then used these cell lines to quantify the expression of another member of the C19MC cluster i.e. miR-520c by real-time PCR (qRT-PCR). Akin to the results obtained for the other members of that cluster high expression was noted only in the same four cell lines with 19q13 rearrangements expressing miR-512-5p, miR-517a, and miR-519a (Figure 3a) whereas a significantly lower expression was seen in the remaining cell lines (*p* = 0.001659; for details see Table 2). Most likely, the exceptionally low expression of all examined members of the C19MC cluster in cell line S121 may result from a deletion of that part of the breakpoint region resulting from the chromosomal translocation the breakpoint of which had previously been mapped at a position between the C19MC and the miR 371-3 cluster. Thus, in all cell lines with breakpoints upstream of the C19MC cluster evidence for an upregulation of four cluster members was obtained. Because of a common regulation of that cluster, no further members were examined.

**Table 2:** Statistical analysis of the qRT-PCR data.

19q translocation	without 19q translocation	microRNA	p-value	d.f.	t
Adenoma	normal thyroid and adenoma	mir371-3p	0.005355	4.48	5.0446
		mir372	2.232e-06	10.996	8.9445
		mir373	0.006122	8.522	3.6176
		mir520c	1.722e-09	10.977	17.9765
Adenoma	adenoma	mir371-3p	0.004623	5.203	4.745
		mir372	0.0001681	7.043	7.2279
		mir373	0.01428	7.9	3.128
		mir520c	4.312e-08	7.978	19.941
Adenoma	normal thyroid	mir371-3p	0.005471	4.159	5.2956
		mir372	0.008832	2.826	6.5061
		mir373	0.02236	4.122	3.5632
		mir520c	0.003133	2.573	10.9875
adenoma cell line	adenoma cell line	mir371-3p	0.004041	4.446	5.4488
		mir372	0.1121	2.4893	2.344
		mir373	0.08229	2.311	2.9573
		mir520c	0.001659	4.029	7.4811

Statistical analysis (t-test, two-tailed) of the expression of miRNAs from the cluster C19MC and miR-371-3 in tissues or cell lines containing 19q13 rearrangements compared to normal thyroid tissue and/or adenomas without 19q13 rearrangements. The data were obtained using statistical software R (www.r-project.org). (d.f. =  degrees of freedom, t =  Student's t-value)

### Thyroid Adenomas with 19q13 Rearrangements Show Upregulation of miRNAs of the C19MC Cluster and the miR-371-73 Cluster

**Figure 4:**
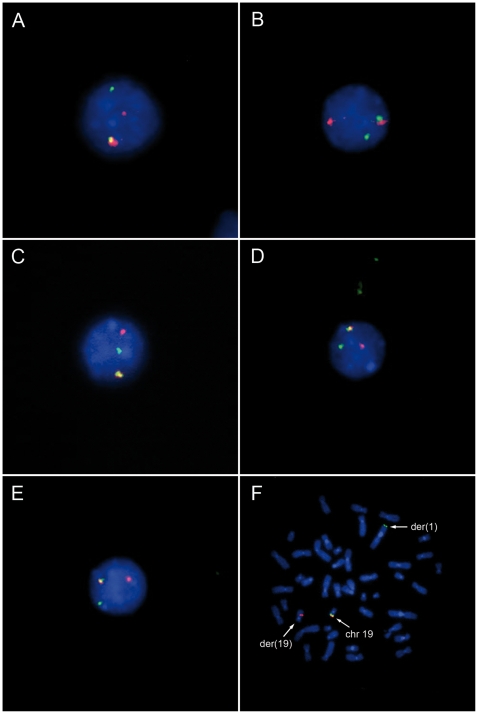
Fluorescence in situ hybridization (FISH) with dual-color, break-apart rearrangement probe (tbpc19). (**A**)**–**(**E**) I-FISH showing 19q13 rearrangements detected using touch-preparations of five thyroid adenomas (**A:** S801, **B:** S814, **C:** S842, **D:** S846, **E:** S849) indicated by separated green (3′-tbpc19) and red signals (5′-tbpc19); (**F**) Metaphase of case S842 with a t(1;19)(q32;q13) after FISH with tbpc19. The 19q13 rearrangement is indicated also by separated signals on der(1) and der(19).

To see if comparable results can be obtained for primary tumors as well we have characterized 70 thyroid nodules by interphase fluorescence in situ hybridization (I-FISH) on cytologic samples obtained prior to cell culturing. The results were usually supplemented by conventional cytogenetics. FISH screening/conventional cytogenetic analysis of the nodules detected five tumors with clonal rearrangements of chromosomal band 19q13 (Figure 4). Akin to the cell lines semi-quantitative RT-PCR revealed an upregulation of 3 miRNAs and again, as further example of that cluster, results were supplemented with qRT-PCR analyses for miR-520. We have shown that all thyroid adenomas with 19q13 rearrangements express significantly higher levels (*p*≤0.003133) of miR-520 than samples without 19q13 rearrangements (adenomas and surrounding thyroid tissue; for details see Table 1) (Figure 3b).

To see if the 19q13 rearrangements also activate the expression of the miR-371-3 cluster we have studied the expression of three members of this cluster in the same samples used before. All tumors with 19q13 rearrangements were shown to express significantly higher levels of miR-371-3p, miR-372, and miR-373 than three samples of surrounding histologically normal thyroid tissue (*p*≤0.02236) and the five cytogenetically normal adenomas (*p*≤0.01428) (Figure 3c). We then quantified the expression of miR-371-3p, miR-372, and miR-373 in the cell lines where comparable results were obtained (Figure 3d). Interestingly, cell line S121 with absent or very low expression of the C19MC cluster members showed a high expression of the miR-371-3 cluster thus further strengthening the idea that in this cell line part of the C19MC cluster is deleted.

### In a Thyroid Adenoma Cell Line the C19MC Cluster Becomes Part of a Pol-II Fusion mRNA

**Figure 5:**
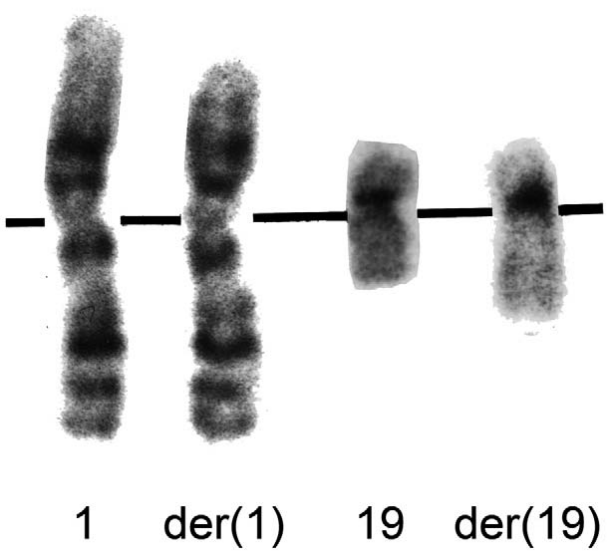
Partial karyotype of cell line S40.2. Partial G-banded karyotype showing chromosome 1 and 19 as well as their derivatives resulting from t(1;19)(p35.2;q13.4).

**Figure 6:**
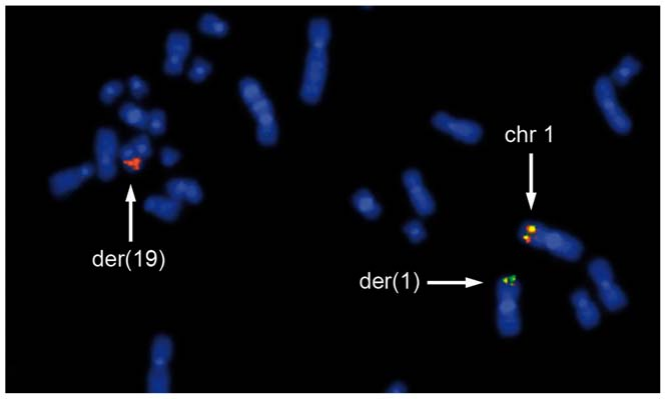
Delineation of *PUM1* breakpoint by metaphase FISH. Part of metaphase of cell line S40.2 after FISH with two overlapping BAC clones RP11-201O14 (green) and RP11-1136E4 (red) both spanning the whole genomic sequence of *PUM1* in 1p35.2. The breakpoint in 1p35.2 is located within *PUM1* indicated by a separation of RP11-201O14 and RP11-1136E4. Because of weak signals of RP11-1136E4 remaining on the der(1) the breakpoint is located within RP11-1136E4 distal to RP11-201O14.

**Figure 7:**
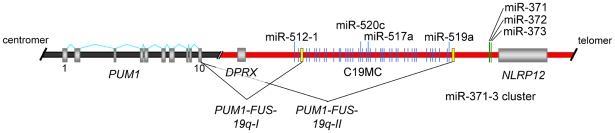
Genomic organization of the fusion gene on the derivative chromosome 1 resulting from a translocation t(1;19)(p35.2;13.4) in cell line S40.2. Detailed schematic overview illustrating the origin of the fusion transcripts *PUM1-FUS-19q-I* (Genbank Accession number GQ334687) and PUM1-FUS-19q-II (Genbank Accession number GQ334688) identified in cell line S40.2. The genomic region of PUM1 in 1p35.2 (horizontal gray bar) fuses after exon 10 of *PUM1* (exons: vertical light gray bars) to the genomic region of *C19MC* in 19q13.4 (horizontal red bar). The two vertical yellow bars indicate 3′-sequences located after exon 1–10 of *PUM1* in *PUM1-FUS-19q-I* and *PUM1-FUS-19q-II*, respectively, both originating from alternative splicing. The fusion transcripts were detected either by 3′-RACE-PCR (*PUM1-FUS-19q-I*) or RT-PCR (*PUM1-FUS-19q-II*) experiments. The quantified miRNAs have been highlighted by their names.

**Figure 8:**
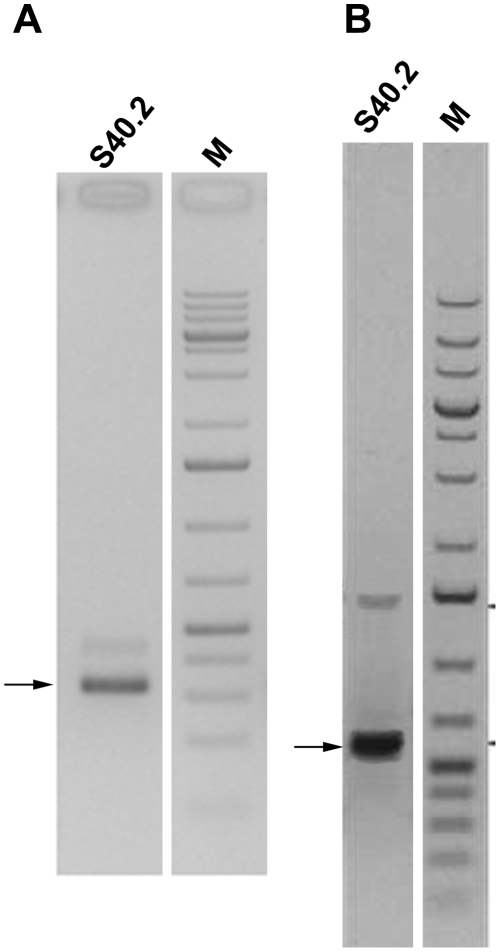
Cloning of fusion transcripts PUM1-FUS-19q-I and PUM1-FUS-19q-II. Fusion transcripts resulting from the t(1;19) in cell line S40.2 were detected by RT-PCR and analyzed by gel electrophoresis. M =  Marker DNA (1 kb+, Fermentas). S40.2 =  Thyroid adenoma cell line S40.2. The arrows point to the corresponding bands that were excised. Isolated DNA was sequenced and analyzed. Weak bands above may represent splice variants. **A**) Transcript PUM1-FUS-19q-I generated with primers Ex9_up and 19_2. **B**) Transcript PUM1-FUS-19q-II generated with primers Ex9_up and 500_Cluster_polyA_I.

**Figure 9:**
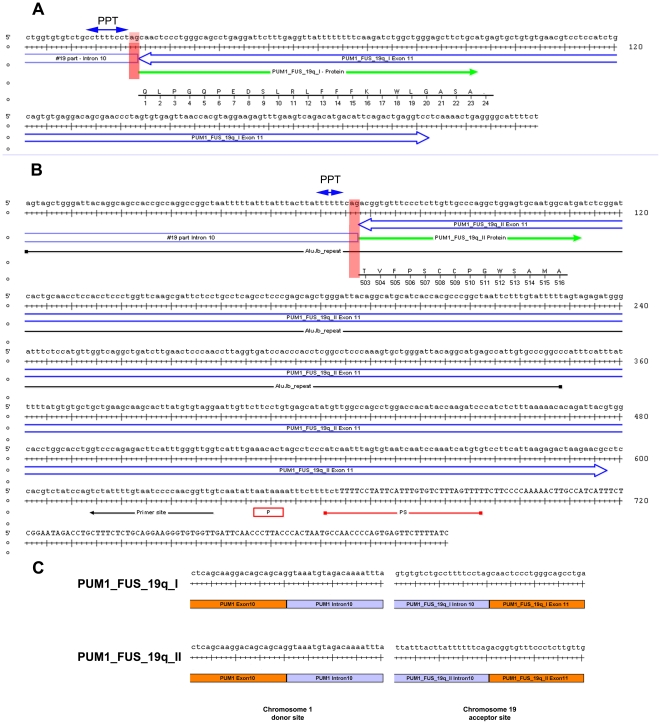
Sequence analysis of the genomic structure of *PUM1-FUS-19q.* Genomic organization of part of *FUS-19q*. Blue double arrows indicate exon 11 of *PUM1-FUS*-19q. The green arrow marks the final part of the fusion protein. The underlying ruler shows the final amino acid sequence. The bases ag (red) correspond to the intron 10 (chromosome 19 part) splice site. Solid blue double arrows indicate the polypyrimidine tract (PPT). **A**) Chromosome 19 derived part of the genomic sequence of *PUM1-FUS-19q-I*. **B**) Chromosome 19 derived part of the genomic sequence of *PUM1-FUS-19q-II*. Black line indicates the Alu–repeat. Site of Primer 500-Cluster_PolyA_I is shown by a black arrow. Red line (PS) and red box (P) indicates predicted poly (A) signals. **C**) Intron 10 splice sites of the two *PUM1-FUS-19q* variants.

In order to further understand the mechanisms involved in the activation of the miRNA clusters one cell line of the 19q13 group has been investigated in more detail. This cell line shows a rearrangement of chromosomal band 19q13.4 resulting from an apparently balanced translocation t(1;19)(1p35.2;q13.4) (Figure 5). By appropriate FISH analyses using BAC (bacterial artificial chromosome) probes the breakpoint on chromosome 1 was mapped within *pumilio homolog 1* (*PUM1*) (Figure 6). *PUM1* encodes a RNA-binding protein and shows a widespread expression in adult tissues. It has 22 exons and spans about 150 kb on chromosomal band 1p35.2 [11], [12]. To further characterize the breakpoint region at the molecular level additional FISH studies were performed allowing to narrow down the 1p35.2 breakpoint to the 6,144 kbp intron 10 of *PUM1*. By the translocation the proximal part of *PUM1* including exons 1–10 was found to be juxtaposed to the miRNA clusters (Figure 7). In addition, we have therefore used 3′RACE-PCR to detect possible fusion transcripts between the proximal part of *PUM1* and sequences from chromosome 19. Among several aberrant transcripts a fusion transcript consisting of exon 1–10 of *PUM1* followed by an ectopic sequence of the chromosome 19 breakpoint region was detected (Figure 8) and sequenced (Genbank Accession number GQ334687). This transcript further confirms that the chromosomal break in this cell line is indeed located within intron 10 of *PUM1*. Accordingly, we have performed RT-PCR experiments using a sequence within exon 10 as the forward primer by which we were able to detect part of one fusion transcript (Figure 8) (Genbank Accession number GQ334688) with a border clearly extending the distal border of C19MC (Figure 7). The 5′ splice site of intron 10 is not completely homologous to the consensus sequence of human introns (Figure 9c)[13]. But the 3′splice sites on either site of the fusion are in line with the conserved consensus sequence and a upstream region known as the polypyrimidine tract (Figure 9a,b). However, from these results it seems reasonable to assume that in the cell line S40.2 both clusters become part of large Pol-II transcript driven by the *PUM1* promoter. An *in silico* analysis of intron 10 of *PUM-1* as well as the chromosome 19 breakpoint region did not reveal obvious sequence homologies pointing to homologous recombination as a mechanism underlying the chromosomal rearrangement seen in that cell line.

## Discussion

Specific structural chromosome abnormalities have turned out to be valuable signposts indicating the position of protein-coding genes with oncogenic potential. Recently, in addition some evidence for a causal association of some chromosomal rearrangements with the activity of microRNA coding genes has been presented [14], [15], [16]. Herein, we were able to show that a highly frequent translocation in benign thyroid tumors i.e. the 19q13.4 rearrangement targets and activates two microRNA clusters in close proximity to the chromosomal breakpoint cluster the expression of which is otherwise almost exclusively confined to embryonic and fetal development. Activation by an ectopic Pol-II promoter may generally be the mechanism by which the translocations activate both miRNA clusters and fits with the apparent “natural” generation of the miRNAs of the C19MC cluster from a large Pol-II driven transcript as witnessed by the results of a recent study [4]. From the histologic analyses performed herein no evidence for invasiveness of the corresponding tumors has been found that by definition would lead to the diagnosis of a follicular carcinoma but this does not rule out a higher risk of these tumors to become malignant. Generally, members of both clusters have been implicated in malignant growth. In human testicular germ cell tumors evidence for an oncogenic potential of miR-373 has been obtained. In these tumors the expression of miR-373 was shown to allow tumorigenic growth in the presence of wild-type p53 [8]. As another example in a recent paper hsa-miR-518c and hsa-miR-373 were among the microRNAs associated with the tumorigenesis of retinoblastomas [17]. In breast cancer, miR-373 and miR-520c promote tumor invasion and metastasis *in vivo* and *in vitro* by the suppression of CD44 [10]. Interestingly, the expression of a miRNA of the C19MC cluster i.e. miR-516-3p recently has been linked to higher aggressiveness of breast cancer as well. Based on a large-scale screen for miRNA expression patterns associated with distant metastasis Foekens et al. [17] were able to show that miR-516-3p belongs to four miRNAs the expression of which is associated with an adverse prognosis in estrogen receptor-positive, lymph node-negative primary breast cancer [18]. Moreover, forced expression of miR-373 leads to a reduction in the nucleotide excision repair (NER) protein, RAD23B, as well as in RAD52 [19] thereby possibly contributing to a higher genome instability.

Based on these data it may be hypothesized that activation of both clusters by chromosomal rearrangements might be not restricted to thyroid tumors. Balanced translocations involving 19q13.4 have also been described in mesenchymal hamartoma of the liver (MHL), a rare benign tumor-like lesion of childhood [20]. Of note, quite recently, genomic amplifications including the C19MC cluster have been detected by array CGH and FISH as recurrent genomic imbalances in an aggressive subgroup in primitive neuroectodermal brain tumors. Functional studies implicated two miRNAs of the cluster i.e. miR-517c and 520 g as oncogenes causally linked to the development of the disease [21]. More generally, breaks of chromosomal band 19q13 have been reported in a variety of human neoplasms. Furthermore, according to the CancerChromosomes/Mitelman database (NCBI) chromosomal band 19q13 belongs to the areas most frequently targeted by chromosomal aberrations at all in the genome. Thus, it remains to be determined whether or not some of these do also target either of the two or both miRNA clusters investigated herein. However, there is ample evidence that within the thyroid epithelium the clonal re-expression of two important “embryonic” miRNA clusters with thousands of potential targets is causally linked to the development of a large subgroup of thyroid adenomas. Effects of individual of these miRNAs with single targets have been associated with human tumors but mechanistically the effects observed are more likely to result from global changes of gene expression than from the de-regulation of single targets of the corresponding miRNAs.

## Methods

### Ethics Statement

The use of human thyroid tumors for this study (including the preparation of the cell lines S270.2 and S290.1) was approved by the local medical ethics committee and followed the guidelines of the declaration of Helsinki. Only samples that were initially taken for diagnostic purposes were secondarily used for the present study. Because the samples were de-identified and were considered as samples normally discarded, the committee felt that there was no specific patient consent necessary.

### Tissue and Cell Lines

All samples were obtained from patients undergoing thyroid resection in the Department of General and Visceral Surgery of the St. Joseph Stift, Bremen (Germany). One piece of each tumor was stored in Hank's solution for cell culture and a second piece was stored in liquid nitrogen for gene expression studies. The cell lines were derived from thyroid adenoma cells as reported previously [22]. Archival RNAs from fetal, placental and testicular tissue were used as controls.

### Cell Culture and Cytogenetic Analyses

Tissue digestion, cultivation of primary cell lines, and cytogenetic analyses were performed according to previously described methods [2], [23]. Before digestion, each sample was touched onto slides to get samples for FISH screening.

### Isolation of RNA, Reverse Transcription and Real-Time PCR (qRT-PCR) Quantification

Total RNA was extracted from tissue as well as from immortalized cell lines using TRIzol (Invitrogen, Karlsruhe, Germany) reagent, or mirVana (Ambion, Woodward, USA) miRNA isolation kit according to the manufacturer's instructions. miRNA (miR-371-3p, miR-372, miR-373 and miR-520c) and *RNU6B* (*RNA, U6 small nuclear 2*; internal control for relative quantification)-specific cDNA were generated from 10 ng of total RNA using the *Taq*Man microRNA RT Kit and the gene-specific RT primers from the *Taq*Man microRNA Assays (Applied Biosystems, Foster City, CA, USA) according to the manufacturer's instructions. The reactions were incubated in a thermal cycler for 30 min at 16°C, 30 min at 42°C, 5 min at 85°C and then stored at 4°C. All reverse transcriptions included no-template controls and minus RT controls (–RT).

Real-time PCR was performed using an Applied Biosystems 7300 Fast Real Time PCR system with miRNA and *RNU6B*-specific probes and TaqMan Universal PCR Master Mix (Applied Biosystems, Foster City, CA, USA). The reactions were incubated in 96-well plates at 95°C for 10 min followed by 40 cycles of 15 s at 95°C and one min at 60°C. All reactions were run in triplicate. Relative quantification (RQ) was calculated using Applied Biosystems SDS software based on the RQ = 2_DDCt 2(–Delta Delta C(T)) method [24]. Ct data were normalized to the internal control, *RNU6B* [25].

### Detection of Fusion Transcripts via 3′RACE-PCR

3′RACE-PCR was performed on cell line S40.2. Total RNA was isolated using RNeasy Mini Kit (Qiagen, Hilden, Germany). cDNA syntheses were carried out with slight modifications following the instructions for the M-MLV reverse transcriptase using oligo(dT) primer as anchor primer (Invitrogen, Karlsruhe, Germany). 3′RACE-PCRs and Nested 3′RACE-PCRs were performed as described in the Gene Racer Kit (Invitrogen, Karlsruhe, Germany) adjusted to the conditions for Go*Taq* Flexi DNA Polymerase (Promega, Mannheim, Germany). Southern Blots were carried out as mentioned by Fehr et al. [26] with *PUM1*-specific probe labelled with digoxigenin-11-dUTP (Roche Diagnostics, Penzberg, Germany). Fragments of interest were excised and extracted with the QIAquick Gel Extraction Kit (Qiagen, Hilden, Germany) and were then cloned into the pGEM-T Easy Vector (Promega, Mannheim, Germany). The plasmid DNA from the clones of interest was isolated via QIAprep Spin Miniprep Kit (Qiagen, Hilden, Germany) and sequenced by Eurofins MWG, Ebersberg, Germany.

### Detection of Fusion Transcripts via RT-PCR

With the program polyadq [27], the possible PolyA-site of the C19MC-cluster was detected. Nearby a primer was designed which was later used together with a *PUM1*-specific primer. Total RNA of S40.2 isolated via TRIzol reagent (Invitrogen, Karlsruhe, Germany) was used for cDNA syntheses as previously described. PCR was done with the Go*Taq* Flexi DNA-Polymerase (Promega, Mannheim, Germany) followed by a semi-nested PCR. Fragments of interest were excised and extracted as described above and sequenced by Eurofins MWG, Ebersberg, Germany.

### Validation of the Fusion Transcript

To confirme the former results, different primers localized within the fusion transcript were utilized. The PCRs were carried out as described above. Fragments of the expected size were excised, extracted and sequenced (see above).

### RT-PCR

miRNA-specific-primers for miR-512-5p, miR-517a and miR-519a were designed, as described by Chen et al. [28]. cDNA was generated from 1 µg total RNA according to Chen et al. [28] with small modifications in stem-loop-primer concentration (5 nM), as well as the PCR-reactions that were modified in annealing-temperature (68°C) and -duration (10 s). The RT-PCR was performed with Go*Taq* Flexi DNA-Polymerase (Promega GmbH, Mannheim, Germany). Elongation was run at 72°C for 15 s.

### Primers

See Table 3.

**Table 3:** Used Primers.

PUM 1		
Primer (Exon)	sequence (5′ -3′)	company
Ex1_Up (Exon 2)	CCCTCAAGAACCAGCTAATCCCAACA	Invitrogen
Ex3_Up (Exon 4)	TTCCTGGGTGATCAATGGCGAGA	Invitrogen
Ex4_Up (Exon 5)	TCCCCGGGCGATTCCTGTCT	Invitrogen
Ex5_Lo (Exon 6)	TCCATCACATCACCCTCCTCCTTCAA	Invitrogen
Ex7_Up (Exon 8)	ACCTAATGCGCTTGCTGTCCA	Invitrogen
Ex8_Up (Exon 9)	GCTCCCGCTGCGTTTGTCC	Invitrogen
Ex9_Up (Exon 10)	CAACAGACCACCCCACAGGCTCAG	Invitrogen
Ex11_Lo (Exon 12)	ATTTCTCGCGCCTGCATTCACTAC	Invitrogen
Ex12_Up (Exon 13)	CCAGTTCTTTCTACGGCAACAACTCTCTG	Invitrogen
Ex14_Up (Exon 15)	AACTGCGGGAGATTGCTGGACATA	Invitrogen
Ex14_Lo (Exon 15)	CCATTATATGTCCAGCAATCTCCCGC	Invitrogen
Ex17_Lo (Exon 18)	CGATGATAAATTGCAAAGACTGGGGC	Invitrogen
Ex19_Up (Exon 20)	TGAGGATAAAAGCAAAATTGTAGCAGAA	Invitrogen
Ex20_Up (Exon 21)	GGAGCCAGGCCAGCGGAAGATC	Invitrogen
Ex21_Lo (Exon 22)	GCCAGTGAGGTCAGCGGGAATG	Invitrogen
3′UTR_Lo (Exon 22)	AATCCAGTAGGCAGTAAACAATCACACC	Invitrogen
5′UTR_Up (Exon 1)	AGAGAGAAGATCGGGGGGCTGAAAT	Invitrogen

cDNA/3′RACE		
AP2	AAGGATCCGTCGACATC(T)17	Invitrogen
Oligo dT	GCTGTCAACGATACGCTACGTAACGGC	Invitrogen
	ATGACAGTG(T)24	
UAP2	CTACTACTACTAAAGGATCCGTCGACATC	Invitrogen
Gene Racer 3′	GCTGTCAACGATACGCTACGTAACG	Invitrogen
Gene Racer 3′Nested	CGCTACGTAACGGCATGACAGTG	Invitrogen
GAPDH2_Up	GTGAAGGTCGGAGTCAACG	Invitrogen
GAPDH5_Lo	AGGAGGCATTGCTGATGAT	Invitrogen

Chromosome 19 miRNA cluster C19MC		
19_1	GGCTGCCCAGGGAGTTGCT	Invitrogen
19_2	GCAGAAGCTCCCAGCCAGATCTT	Invitrogen
19_3	CTAGGGTTCGCTGTCCTCACACTGC	Invitrogen
500-Cluster_PolyA_I	CAACCGTTGGGGATTACAAAATAGA	Invitrogen

Stem-loop-Primers		
miR-371-5p a sl	GTCGTATCCAGTGCAGGGTCCGAGGTAT	Invitrogen
	TCGCACTGGATACGACACACTC	
miR-371-5p sl	GTCGTATCCAGTGCAGGGTCCGAGGTAT	Invitrogen
	TCGCACTGGATACGACAGTGCC	
miR-372 sl	GTCGTATCCAGTGCAGGGTCCGAGG	Invitrogen
	TATTCGCACTGGATACGACACGCTC	
miR-373 sl	GTCGTATCCAGTGCAGGGTCCGAGGTAT	Invitrogen
	TCGCACTGGATACGACACACCC	
miR-512-5p sl	GTCGTATCCAGTGCAGGGTCCGAGG	Invitrogen
	TATTCGCACTGGATACGACGAAAGT	
miR-517a sl	GTCGTATCCAGTGCAGGGTCCGAGG	Operon
	TATTCGCACTGGATACGACACACTC	
miR-520c-3p sl	GTCGTATCCAGTGCAGGGTCCGAGGTAT	Invitrogen
	TCGCACTGGATACGACACCCTC	
miR-520c-5p sl	GTCGTATCCAGTGCAGGGTCCGAGGTAT	Invitrogen
	TCGCACTGGATACGACCAGAAA	

Forward and Reverse Primers (PCR)		
miR-371-3p fw	ACCGCTAAGTGCCGCCATCTTTTG	Invitrogen
miR-371-5p fw	GCCGCCACTCAAACTGTGGGG	Invitrogen
miR-372 fw	GGTCATAAAGTGCTGCGACATTTG	Invitrogen
miR-373 fw	TTCATGAAGTGCTTCGATTTTGG	Invitrogen
miR-512-5p fw	AGTCTACACTCAGCCTTGAGGGCA	Invitrogen
miR-517a fw	CGGCGGATCGTGCATCCCTTTA	Operon
miR-519a fw	CCGGCTAAAGTGCATCCTTTTAG	Invitrogen
miR-520c-3p fw	GCCGCCAAAGTGCTTCCTTTTAG	Invitrogen
miR-520c-5p fw	ACCGCTCTCTAGAGGGAAGCAC	Invitrogen
Reverse Primer	GTGCAGGGTCCGAGGT	Operon

Primer sequences for PCR and cDNA synthesis.

### Fluorescence In Situ Hybridization (FISH)

Interphase FISH (I-FISH) analyses were performed on touch-preparations of thyroid tumors. For detection of 19q13.4 rearrangements a dual-color, break-apart rearrangement probe (PanPath, Budel, Netherlands) referred to as tbpc19 (thyroid breakpoint cluster 19q13) was used. The rearrangement probe is a mixture of two probes located distal (3′-tbpc19; labeled by Alexa Fluor 488) and proximal (5′-tbpc19; labeled by AlexaFluor 555), respectively, of the common breakpoint-cluster region in 19q13.4 in benign thyroid lesions. 10 µl of the break-apart probe were used per slide. Co-denaturation was performed on a Mastercycler gradient (Eppendorf, Hamburg, Germany) for 3 min at 80°C followed by overnight hybridization in a humidified chamber at 37°C. Post-hybridization was performed at 61°C for 5 min in 0.1xSSC. Interphase nuclei were counterstained with DAPI (0.75 µg/ml). Slides were examined with a Axioskop 2 plus fluorescence microscope (Carl Zeiss, Göttingen, Germany). Images were captured with an AxioCam MRm digital camera and were edited with AxioVision (Carl Zeiss, Göttingen, Germany). For each case 200 non-overlapping nuclei were scored. Co-localized signals (green/red) indicate a non-rearranged breakpoint region, whereas separated green and red signals indicate a rearrangement of the chromosomal region 19q13.4. Metaphase-FISH with tbpc-19 on case S842 was performed as described above for I-FISH on touch-preparations. Treatment of metaphases was carried out as described by Kievits et al. [29].

For determination of the breakpoint on chromosome 1 FISH was performed on metaphase preparations of the cell line S40.2. As probes two overlapping clones RP11-1136E4 (Genbank Accession number AQ707626 and AQ733864) and RP11-201O14 (Genbank Accession number AL356320.8) (imaGenes, Berlin, Germany) both spanning the whole genomic sequence of *PUM1* were used. DNA was isolated using Qiagen Plasmid Midi Kit (Qiagen, Hilden, Germany). 1 µg of isolated plasmid DNA was labeled by nick translation (Roche, Mannheim, Germany) either with digoxigenin-11-dUTP (RP11-201O14) or biotin-16-dUTP (RP11-1136E4). Treatment of metaphases and subsequent FISH experiments were carried out as described previously by Kievits et al. [29] with exception for co-denaturation and post-hybridization which were performed as described above.

### Statistical Analysis

Results are presented as the mean ± standard error (SE). Statistical comparisons were performed by a nonpaired Student's t -test. A p-value of less than 0.05 was considered significant.

### Data Deposition

The complete fusion transcript sequences has been deposited in GenBank, PUM1-FUS-19q-I (Genbank Accession number GQ334687) and PUM1-FUS-19q-II (Genbank Accession number GQ334688).
